# Supplementary material for: Are carbon emissions trading and green financial instruments synergistic? -Comprehensive quantitative research based on content analysis
Source: PLoS One. 2024 Mar 7;19(3):e0298601. doi: 10.1371/journal.pone.0298601 (PMC10919601; doi:10.1371/journal.pone.0298601)
Supplement: S1 Data — (ZIP) [file pone.0298601.s001.zip › Data/地级市Z_F工作报告.zip!/┴╔─■/╙¬┐┌╩╨/└┤╫╘╣1⁄2╓┌║┼í╛┬φ┐╦╩2╛▌═°í┐_2015.docx]

各位代表：

　　现在，我代表市政府向大会报告工作，请予审议，并请市政协委员和列席会议的同志提出意见和建议。

　　一、2014年工作回顾

　　过去的一年，在省委、省政府和市委的正确领导下，在市人大、市政协的监督支持下，市政府组织带领全市人民，积极应对严峻复杂的经济形势，坚定信心，保持定力，统筹推进稳增长、促改革、调结构、惠民生、防风险各方面工作，保持了经济平稳健康发展、社会和谐稳定的良好局面。

　　预计全年实现地区生产总值1610亿元，增长6.5%；固定资产投资1160亿元，下降8.6%；公共财政预算收入160.4亿元，下降13%；社会消费品零售总额436亿元，增长12%；城镇常住居民人均可支配收入28325元，增长9.5%；农村常住居民人均可支配收入12800元，增长12%；城镇登记失业率2.45%；居民消费价格指数增长1.7%。

　　（一）全力以赴稳增长，全市经济平稳健康发展

　　认真贯彻落实国家振兴东北的一系列重大举措和稳增长的各项措施，新一轮振兴政策的带动效应开始显现。积极配合省政府“6+4”重大课题和重要专项工作调研，摸清底数，找准问题，采取措施，促进全市经济稳步增长。

　　加快县域工业化、城镇化和农业现代化步伐，农业和农村经济稳步发展。农业增加值完成115亿元，增长5%。

　　粮食生产克服严重旱灾影响实现灾年稳产，总产量达6.7亿公斤；水稻高产创建平均亩产和最高单产连续6年居全省首位；水果减产不减收，蔬菜、水产品、肉蛋奶产量比上年均有增长。“米袋子”、“菜篮子”保障能力进一步提升。

　　现代农业加快发展，新建设施农业小区1.2万亩、蔬菜基地1.1万亩，新增“三品”认证30个、认定面积34万亩；“营口对虾”等10个农产品品牌成功晋升国家和省级名牌，盖州市获批辽宁省“一县一业”示范县，鲅鱼圈区获批省级海蜇出口基地，全市农产品出口增长20%。精品农业引领现代农业发展的能力不断增强。

　　龙江福大豆和津大肥业2个投资10亿元以上农产品加工项目投产运行；农业综合机械化水平进一步提升，全年节本增效2.5亿元；新增农民专业合作社194家，规范110家。农业增效、农民增收的基础更加坚实。

　　狠抓以企业为核心的经济运行，工业经济实力持续增强。规模以上工业增加值完成838亿元，增长7%。

　　加大帮扶力度，促进企业平稳运行。组织企业参加各类展览会、展洽会，积极帮助企业开拓市场；不断加强要素保障，进一步完善五矿营钢、忠旺铝业等企业要素配套，保证企业要素需求；支持中小微企业发展，在融资担保和争取专项资金等方面加大扶持力度，促进了企业发展壮大。全年新增规模以上企业100户、主营业务收入超10亿元企业7户。

　　坚持围绕转型升级扩增量，全年开（复）工投资千万元以上工业项目567个，其中亿元以上项目249个。信义玻璃、无限极健康食品等一批重大项目竣工投产，新项目的产能释放作用不断增强。

　　加快产业集群发展，全市17个工业产业集群主营业务收入增长7%；镁产品及深加工、钢铁及深加工和石化产业集群分别达到890亿、570亿和530亿元。产业集群的集聚度和丰厚度进一步提高。

　　积极推进科技创新和淘汰落后产能，实施重点技术改造项目200个，淘汰落后印染产能900万米、电熔镁产能6.6万吨。加快工业化和信息化融合步伐，规模以上制造业企业普遍采用了计算机辅助技术。高新技术产品增加值占规模以上工业增加值比重达到40%。创新驱动正在成为经济发展的强劲力量。

　　全面实施加快发展服务业“四年行动计划”，服务业对经济发展的贡献率得到提升。全年开（复）工投资千万元以上服务业项目591个，服务业增加值实现660亿元，占地区生产总值比重达到41%。

　　城市中央商务区和服务业集聚区建设成效显著，9个省级服务业集聚区主营业务收入560.3亿元，增长20%；万达广场已经封顶，万隆广场等一批重大服务业项目投入运营。

　　海滨、温泉、沟域等特色旅游发展后劲持续增强。北海国家海洋公园向游人开放，河海龙湾温泉度假酒店、亲和源温泉康复医院等一批项目投入运营。全年旅游总收入260亿元，增长20%。我市被评为“中国十大特色休闲城市”。

　　全年新增贷款192.4亿元，多渠道融资330亿元。辽宁大族冠华、中镁控股在“新三板”上市，抚顺银行营口分行、营口农商银行挂牌营业。金融业发展保持强劲态势。

　　物流业提质升级成效明显，增加值实现200亿元，增长7.5%。营口港现代物流等服务平台投入使用。新晋全国A级物流企业46家、省级现代物流示范企业15家。

　　营口汽保产品、大宗商品等电子交易平台运行平稳，阿里巴巴（营口）产业带上线运行，营口兴隆等商贸企业网上商城加快建设。电子商务成为现代服务业发展的新亮点。

　　（二）深化改革开放，经济社会发展活力不断增强

　　重点领域改革取得新进展。行政审批制度改革和简政放权全面推进，取消行政审批等事项148项，下放271项。工商登记制度改革稳步推进，全市新登记企业、公司制企业分别增长49.8%和59.6%。支持实体经济发展，暂免征收部分小微企业增值税和营业税、减半征收小微企业所得税1.5亿元，受益企业4256户；取消、减免行政事业性收费和基金，减轻企业负担1.2亿元；通过扩大“营改增”试点范围，直接减税6亿元，受益企业2016户。国库集中支付等财政改革扎实推进，实施综合治税，财税管理不断加强。政府债务甄别基本结束，债务管理进一步规范。农村综合改革扎实推进，完成土地确权登记颁证16万亩，集体林权登记发证率97.6%。在全省率先完成工业企业生产性服务业主辅分离任务。户籍改革、公车改革稳步推进。改革红利的释放效应持续增强。

　　港城联动实现新突破。积极融入国家“一带一路”战略，辽鲁陆海货运大通道开航，营满欧国际货运直达班列开通，营口在亚欧大陆桥东线的“桥头堡”地位凸显。综合保税区可研报告通过专家论证，中韩自贸示范区申办工作正式启动，鲅鱼圈港口物流贸易园区获评“全国优秀物流贸易园区”。全年港口货物吞吐量完成3.3亿吨，增长3%；集装箱运量560万标箱，增长5.6%。港城联动、互联互通的大发展格局正在形成。

　　对外开放取得新成果。全面实施招商选资，全市实际利用外资14亿美元，增长5.3%；引进内资422亿元，增长20%。全年开（复）工投资千万元以上项目1170个，其中亿元以上项目602个。德国“萨固密”一期等一批重点项目竣工投产。全年外贸出口44亿美元，增长0.7%，出口对经济发展继续发挥支撑作用。

　　园区建设和发展取得新成效。扎实开展“园区建设年”活动，六个重点园区基础设施建设投资达46.8亿元，园区承载能力不断增强；开（复）工投资千万元以上项目数量占全市的52.3%，规模以上工业增加值占全市的32.6%，公共财政预算收入占全市的44.6%，园区对全市经济贡献率不断提升。

　　（三）加强城乡建设和管理，城乡面貌不断改观

　　城市发展规划进一步完善。以“一带三轴”为核心的城市发展总体规划和控制性详细规划、各专项规划编制基本完成，城乡规划体系基本形成。

　　城市管理不断强化。加大对违法建设、交通秩序、广告牌匾等专项整治力度，市容市貌有了较大改观。扎实开展土地审计，土地管理更加规范。智慧城市建设加快推进，完成城市基础数据和公共信息共享平台专项设计，城市管理步入精细化、科学化轨道。

　　城市功能不断完善。兰旗机场各项设施全面建成，通航在即；华能营口仙人岛热电项目开工建设；城市垃圾处理厂主体建设完工；新华路、建设街等12条街路改造工程全部完成；大旱河、大清河区段清淤防洪工程完成；大辽河主城区段防洪治理主体工程完工，防洪能力达到百年一遇标准。基础设施建设的不断加强，为我市经济社会发展和人民生活质量持续提升打下了坚实基础。

　　“青山、碧水、蓝天”三大工程深入实施。义务植树650万株，营造经济林2.9万亩，“国家级森林城市”创建通过验收。治理生产矿45处、面积973.6亩，闭坑矿5处、面积856亩。城镇污水处理设施进一步完善，日处理污水能力达40万吨；气化营口、区域一体高效供热、大气监控预警等六项工程有序推进，蓝天工程项目全部完成，生态环境、人居环境明显改善。

　　（四）倾力保障和改善民生，社会事业全面进步

　　过去一年，全市重点民生工程投入37.2亿元，是历史上投入最大的一年。外环路外迁改造工程已实现简易通车；完成主城区桂丰园、志诚等57个老旧小区提升改造；安置回迁居民14452户，主城区超期回迁问题基本解决；完成市区西部管网集中供热工程，新增供热能力1550万平方米，主城区基本实现全域集中供热；宽带提速、4G网络建设和549个行政村的亮化工程、210公里的农村道路改造、1440户农村危房改造等重点民生工程基本完成，群众关心关注的一些热点难点问题得到解决。

　　就业和社会保障能力不断增强。新增实名制就业5.26万人。新增城镇基本养老保险5.5万人、城镇基本医疗保险2.1万人。新农合参合率达98.8%。城乡低保保障标准分别提高10%和11%，实现应保尽保。新型农村养老保险和城镇居民养老保险制度改革全面完成，农村和城镇居民养老保险合并实施。深入推进东部振兴战略，移民扶贫296户，东部山区农民人均年收入增加1020元。

　　社会事业全面进步。积极推进城乡教育均衡发展，提前完成省定50所义务教育学校标准化建设任务，基础教育强县（区）建设走在全省前列。中小学规范办学成果显著，中职毕业生就业率达93%。基本公共卫生服务均等化工作全面实施，县级公立医院改革有序推进，新农合管理经验在全国推广；新建市康复医院，填补了我市医疗服务领域空白。群众文化生活丰富多彩，我市荣获“中华诗词之市”称号。实施文物保护工程，13处文物古迹晋升省级文物保护单位；实施文化产业培育和引领工程，艺术剧院创作的《梦回摇篮》等4个项目列入了国家重点文化产业项目库。群众体育蓬勃开展，竞技体育水平不断提高。在省第十二届运动会上，我市金牌总数列全省第4位。城市文明水平持续提升。

　　社会治理不断加强。“六五”普法深入实施，我市被评为全国“六五”普法中期先进市。加强平安营口建设，强化打防管控能力，人民群众安全感不断提升。健全信访工作长效机制，社会保持和谐稳定。不断加大安全生产专项整治力度，全市安全生产形势持续平稳。深化食品药品监管体制改革，强化监管措施落实，人民群众饮食用药安全得到切实保障。

　　（五）深入开展党的群众路线教育实践活动，政府自身建设进一步加强

　　作风建设更加强化。按照中央、省委和市委的部署，以为民务实清廉为主题，深入排查“四风”问题，着力抓好整改落实、建章立制等关键环节，促进作风建设常态化、长效化。中央巡视反馈意见及市政府班子对照检查出的78个问题全部整改落实，集中开展清理福利企业、机关办协会、中介组织等专项整治，一些关系群众切身利益的现实问题得到解决，行风、政风明显转变，办事效率进一步提高。

　　依法行政更加严格。认真学习贯彻党的十八届四中全会精神，努力提高依法行政水平。自觉接受人大依法监督和政协民主监督，积极听取各民主党派、工商联、无党派人士和各人民团体的意见，人大代表建议、政协提案全部办复。依法开展行政复议、行政应诉，保障公民与法人的合法权益；规范行政、决策程序，坚持土地决策、规划联审等集体决策制度，依法行政、依规办事的自觉性显著增强，科学决策、民主决策、依法决策水平不断提高。

　　廉洁从政更加自觉。严格执行中央“八项规定”和省委、市委“十项规定”，加强惩防体系“五大系统”建设。全年市本级“三公”经费下降15%。加强行政监察、审计监督和廉政风险防控机制建设，促进行政职权公开公正透明。

　　双拥共建、国防教育、民兵预备役和人民防空工作扎实推进；人口和计划生育、统计、新闻出版、国家安全、民族宗教、外事侨务、史志、档案、气象、地震等工作不断加强；慈善、老龄、妇女儿童、残疾人、未成年人等各项事业取得新成绩。

　　各位代表：去年，我市一些主要经济指标没有完成年初确定的目标任务，其主要原因：一是我市经济发展处于增长速度的换挡期、结构调整的阵痛期、前期刺激政策的消化期，在全国性的大环境、大背景下，我市这样重化工业比重较高、传统产业份额较重的城市，经济增长速度下滑，是结构调整的必然，是经济规律作用的结果。二是受宏观经济形势影响，部分企业运转困难，效益下滑，导致财税减收；落实国家和省“营改增”扩面、小微企业减税让利等政策性因素，影响全市公共财政预算收入下降2.8个百分点；我们坚持实事求是组织财政收入的原则，确保财政收入数据真实。这些因素致使财政收入有所减收。三是外部宏观环境复杂严峻，市场预期不足，投资意愿下降，投资对经济发展的拉动作用减弱。四是主动转变发展理念，抓当前，利长远，提高经济发展质量，为我市经济持续健康发展创造条件，打牢基础。

　　主要经济指标虽然增速回落，但产业结构、需求结构、分配结构、投资结构均出现了积极的变化；财政收入质量和收入结构显著改善，实体经济税收增长4%，可用财力没有减少；居民收入增速持续高于地区生产总值增速；民生持续改善，劳动力需求旺盛，就业压力减弱，居民消费价格持续平稳。

　　2014年，营口经济发展的基本面没有改变，经济总量在全省的位次没有改变，健康向上的发展势头没有改变，群众生活不断改善的态势没有改变。

　　各位代表：过去一年所取得的成绩来之不易，这是省委、省政府和市委正确领导的结果，是市人大、市政协和社会各界监督支持的结果，是全市人民共同奋斗的结果。在此，我代表市政府，向辛勤工作在各条战线的同志们，向各级人大代表、政协委员、各民主党派、工商联、无党派人士、各人民团体，向离退休老领导、老同志，向驻营解放军、武警部队官兵和中省直单位，向所有关心、支持营口建设发展的同志们、朋友们，表示衷心的感谢并致以崇高的敬意！

　　在看到成绩的同时，我们也清醒地认识到，经济社会发展和政府工作中的一些矛盾和问题还没有很好的解决。经济结构、产业结构、产品结构还有待进一步调整优化；创新环境、创新体系、创新能力亟待提升；财政支撑能力弱、债务负担重的问题还没有很好解决；环境保护、节能减排的任务十分艰巨；与人民群众生活息息相关的民生问题还没有解决到位；一些干部对经济发展新常态还不适应，存在不愿作为、不敢作为、不会作为的问题，公务员队伍建设还有待进一步加强。这些问题，我们要采取有力措施认真加以解决。

　　二、2015年的主要任务

　　2015年是全面深化改革、推进依法治国的关键之年，是全面完成“十二五”规划的收官之年，也是深入实施新一轮东北振兴发展战略的开局之年，做好今年政府各项工作意义重大。当前，我国经济发展进入新常态，这是中央审时度势做出的重大战略判断。我们要深刻认识新常态下速度变化、结构优化、动力转换的新特点，准确把握新常态的发展新趋势，主动适应新规律、新要求，积极作为，抢占振兴发展的先机。

　　适应新常态，实现新发展，既面临新考验、新挑战，更蕴含着新机遇、新动力。中央新一轮支持东北振兴发展战略的实施，为我市提供了最独特、最直接的重大战略机遇；国家“一带一路”重大战略的实施，为我市融入中蒙俄经济走廊，促进更高水平、更宽领域的对外开放，提供了更加广阔的发展空间。经过几年的努力，历史积累的一些矛盾和问题逐步化解，发展质量显著提升；多年来的大量投入，城市功能更加完善，园区基础设施配套更加完备，承载能力明显增强，为我市的全面振兴积蓄了潜力，夯实了基础；康辉石化、无限极、忠旺铝材等一批重大项目的产能正在释放，新的经济增长点正在形成；更为重要的是，经过连续几年来应对严峻复杂的经济形势，我们的干部队伍得到了锻炼，积累了经验，增强了自信，这是我们克服困难、战胜挑战的可靠保障。

　　适应新常态，实现新发展，既要抓好当前稳增长、促改革、调结构、惠民生、防风险的各项工作，又要着眼长远提质增效的各项措施，坚持创新驱动、改革驱动、市场驱动、开放驱动共同发力，力求以较少的要素投入、较小的资源环境代价，获取更大的经济效益，使营口成为资源要素聚集区、互联互通枢纽区、开发开放热点区，把营口老工业基地的发展潜力充分释放出来。

　　适应新常态，实现新发展，必须始终坚持以发展为第一要务不动摇，始终保持旺盛的发展激情，聚精会神干事业，扎扎实实促发展，努力实现营口更高水平、更高质量的全面振兴。我们坚信，有省委、省政府和市委的坚强领导，有市人大、市政协的监督支持，有全市上下发奋图强、锐意进取的强烈愿望，营口的发展一定会更好。

　　今年政府工作总的要求是：全面贯彻党的十八大和十八届三中、四中全会及中央经济工作会议精神，深入学习贯彻习近平总书记系列重要讲话精神，按照省经济工作会议和市委十一届九次全会的部署，坚持稳中求进总基调，主动适应经济发展新常态，以提高经济发展质量和效益为中心，以贯彻落实《国务院关于近期支持东北振兴若干重大政策举措的意见》为总抓手，把转方式调结构放在更加突出的位置，保持经济运行在合理区间，深化改革，扩大开放，突出创新驱动，强化风险防控，保障改善民生，促进经济平稳健康发展和社会和谐稳定，努力开创营口老工业基地全面振兴的新局面。

　　综合考虑多方面因素，建议今年我市经济社会发展主要预期目标为：地区生产总值增长6.5%，固定资产投资增长7%；公共财政预算收入与上年持平，社会消费品零售总额增长11.5%，城镇常住居民人均可支配收入增长8%，农村常住居民人均可支配收入增长10%。居民消费价格指数涨幅和城镇登记失业率均控制在3%以下。

　　（一）转变发展方式，提高发展质量

　　新常态的核心是要有质量、有效益、可持续的发展。要坚持以稳增长为首要任务，准确把握新常态下转方式调结构的方向和重点，促进经济发展提质增效升级。

　　围绕现有企业运行抓调整、促转型，推动工业经济向中高端迈进。现有企业是全部经济工作的基础，稳增长首先要稳企业。要坚持一企一策，引导正常生产企业优化产品结构，提高经济效益，为稳增长多做贡献；积极帮助产品有市场、技术含量高、发展前景好的停产、半停产企业恢复生产，盘活存量；加大中小企业扶持力度，开展“千企转型升级行动”，推动1000户中小企业技术改造和新产品开发，以产品升级带动产业优化转型；淘汰一批高耗能、高污染、高排放、低效益、低产出的落后产能。坚持做优增量，加快推进工业项目投达产，信义玻璃等100个项目当年净增产值100亿元以上。全市新增规模以上企业60户，规模以上工业增加值增长7%左右。

　　围绕优势抓三产，促进传统服务业提档升级、现代服务业快速发展。我市的服务业有基础、有条件，比较优势突出，关键是充分发挥优势，充分释放潜力。要加快服务业集聚区建设，全年主营业务收入达到634亿元；充分发挥温泉旅游的战略牵动作用，坚持商旅联动，强化温泉资源管理，促进温泉旅游项目向高端化发展，推动旅游业整体水平不断提升；加快金融创新步伐，组建营口市诚信担保集团，支持港口物流金融体系建设；推进中小企业在“新三板”挂牌，力争金辰机械年内成功上市；抓好台湾商业综合体等156个重大服务业项目建设，加快推进物流业信息化、标准化建设，积极打造电子商务大厦等电商平台，推进阿里巴巴（营口）跨境产业带等电商项目建设。稳定房地产市场，优化产品结构，加强配套设施建设，促进房地产业平稳健康发展。积极适应消费模式新变化，培育壮大消费热点，增强消费对经济增长的基础作用。服务业增加值占地区生产总值比重达到42%。

　　围绕农业增效、农民增收，大力发展现代农业。加快县域工业、服务业发展，提升县域经济整体水平。抓好“米袋子”、“菜篮子”工程，稳定粮食生产，巩固扩大优质水稻高产创建成果，打造粮食生产示范区；发展设施水果和蔬菜，新栽果树250万株、新建蔬菜基地5000亩。加快农业产业化发展，新开工投资千万元以上项目15个。推进标准化生产，新增“三品”认证20个、认定面积10万亩。加强农田水利基础设施和气象灾害监测预警体系建设，增强农业综合生产和防灾减灾能力。积极推广农业机械化，农作物耕种收综合机械化水平达到77.7%。加大东部振兴战略实施力度，促进区域开发与精准扶贫相结合，不断提高山区群众生产生活水平。

　　围绕创新驱动抓转型、促升级。设立新兴产业发展专项基金，壮大新兴产业规模，争取列入省设立的国家产业转移示范区。加强科技创新体系建设，深入开展百家科技企业服务行动，积极参与创建沈大自主创新示范区，加大先进适用技术研发和引进力度，促进科技成果转化。瞄准“工业4.0”，开展工业化和信息化融合试点，推动企业由生产制造型向生产服务型转变。高新技术产品增加值占全市规模以上工业增加值比重达到42%。

　　适应新常态，实现稳增长，必须把政府工作重心放在营造创新环境、凝聚创新人才、维护创新规则、强化创新激励上，让创新的活力充分迸发、创新的潜力充分释放，让创新成为转方式调结构、提质增效的强劲力量。

　　（二）深化改革，不断增强发展活力

　　适应新常态，增强市场驱动，必须加快改革步伐，为创新开辟道路、为竞争搭好舞台、为开放营造空间，汇聚内生发展的强劲动力。

　　深入推进行政审批制度改革。坚持放管结合，进一步加大简政放权力度，清理、调整一批行政审批事项，提升市（县）区政府承接审批事项的能力；全面清理行政权力，加快推进权力清单、责任清单和负面清单制度建设；实施“三证合一”登记制度改革，最大程度实现便利化，营造宽松平等的准入环境。

　　深化国资国企改革。引进社会资本参与国有企业经营，大力发展混合所有制经济。全面推进经营性国有资产集中统一监管，实现国有资产监管全覆盖，保值增值。整合行政事业单位固定资产，改造便民惠民场所，实现国有资产效益最大化。争取试点示范重大政策，力争在解决国企遗留问题、工业化和信息化融合发展试点、东北地区电子商务试点等方面先行先试。

　　深化财税体制改革。落实税收保障办法，实施综合治税，强化税收征管，严格税收纪律，确保应收尽收。健全预算体系，规范预算管理。加强专项资金监管，深化国库管理改革，全面推进乡镇国库集中支付和非税收缴改革试点。做好“营改增”扩围工作，加快推进政府购买公共社会服务改革。建立健全政府债务管理制度和风险预警处置机制，将政府负有偿还责任的存量债务纳入预算管理，严控债务风险，坚决杜绝举新债铺新摊子。

　　积极探索投融资体制改革。加快营口港口金融创新试验区发展，推进北方金融产品交易中心和运营管理中心审批建设。推行政府和社会资本合作（PPP）等多种投融资模式。巩固并创造良好的金融生态环境，加大金融扶持中小微企业力度，增强金融运行效率和服务实体经济的能力。

　　大力促进非公有制经济发展。创新扶持模式和政策，发展壮大民营经济，推进民营企业集团建设，选择10户民营企业进行现代企业制度试点。积极争取国家中小城市综合配套改革和民营经济改革试点。

　　继续深化农村改革。扩大土地确权登记颁证试点范围，新增确权登记30万亩。加快新型农业经营体系建设，新增、规范农民专业合作社各100家、新增家庭农场20个。推进农村土地适度规模经营，新增土地流转面积3万亩。

　　（三）扩大开放，积极打造培育新增长点

　　适应新常态，增强开放驱动，必须实施更加积极的开放战略，以开放引领产业和技术创新，以开放拓展国内国外两个市场，不断创造发展新优势、新空间。

　　积极融入国家“一带一路”战略，努力打造经济发展新引擎。坚持港城联动，着力促进互联互通，打造辽宁沿海经济带重要支点。充分发挥港口牵动辐射作用，积极促进营口与沈阳经济区各市一体化发展。加强港口基础设施建设，完善陆港布局；巩固内贸航线领先地位，加快培育开发国际航线。港口货物吞吐量达到3.4亿吨，增长3%；集装箱运量590万标箱，增长5.3%。推进综合保税区申办和中韩自贸示范区论证规划工作，加快现代物流业发展步伐，促进货值落地、税收落地。加快辽鲁陆海货运大通道和营满欧大陆桥发展，融入中蒙俄经济走廊建设，打造新亚欧大陆桥“桥头堡”，努力建设东北亚物流之都。

　　坚持围绕项目抓发展，促进投资稳定增长和结构优化。项目建设是经济工作的重中之重。要加大中央新一轮东北振兴政策举措的落地力度，加快推进与央企、民企和沪企合作项目以及鲅鱼圈鞍钢二期等重点项目开工建设；营口万达广场年内部分投入运营，沈鼓集团营口生产基地等一批重大项目竣工投产。全年开（复）工投资千万元以上项目774个，其中亿元以上项目431个，让中央振兴东北的一系列重大举措在营口落地生根，开花结果。

　　坚持围绕产业结构转型升级抓招商，加大招商选资力度。严格执行国务院〔2014〕62号文件，充分发挥比较优势，充分利用现有资源，转变招商方式，实施“嫁接式”招商、点对点招商，积极承接国内外产业转移，重点引进用地集约、附加值高、无污染、低耗能、有利于产业优化的项目。

　　坚持围绕项目抓园区，进一步明确园区功能定位。以项目投达产为目标，完善园区建设，促进产能释放。加大土地清理力度，提高投资强度，集约节约利用土地。加快园区去行政化改革步伐，增强园区发展的内生动力。

　　优化贸易结构，促进对外贸易稳定增长。积极引导内贸企业加大国外市场开发力度，扩大地产商品出口。推动外贸出口基地提档升级，力争营口汽保、营口管件晋升为省级出口基地，耐火材料晋升为国家级出口基地。加强口岸建设，努力构建“大通关”格局，促进通关和对外贸易便利化。全年外贸出口增长6%左右。

　　（四）加强城市建设和管理，提升城镇化水平

　　坚持以创促建，全面提高城市建设和管理水平，努力创建全国文明城市。

　　严格执行城市发展规划，坚持一张蓝图绘到底。明确城市区域功能定位，编制和完善城市供水、供气、供热、地下综合管廊等专项规划，基本实现市政公用基础设施专项规划全覆盖。

　　坚持高水平改造老城区、高质量建设新城区、高档次发展小城镇，统筹推进城乡建设。加大棚户区改造力度，妥善安置动迁回迁居民。实施农村公路、农村客运站亭等农村交通设施建设工程，推进营仙路建设，适时启动快速公交工程，完成营盖公路改造，兰旗机场实现通航。积极稳妥推进城镇化建设，坚持以人为核心，加大近郊农村、城中村、园中村改造力度，完善城镇基础设施，提高基本公共服务供应能力，使人民群众在城镇化进程中生活得更舒适、更幸福。

　　加快智慧城市建设，打造高效、便捷、安全城市。加快推进协同办公一体化服务平台、网上审批一站式服务平台建设，实现政府办公信息化，提升电子政务水平，让群众充分享受智慧服务带来的便利。实施道路交通、违法建设等专项整治，提高精细化管理水平。深入推进宜居乡村建设，创建省级示范村19个，全面完成农村环境综合整治任务，努力打造美化、亮化、净化的宜居营口。

　　加强生态保护，改善生态环境。提高森林城市建设水平，造林1500亩、义务植树200万株，完成矿山生态治理856亩。强化流域内涉水污染源环境监管，充分发挥污染治理各项工程持续治污能力，进一步改善河流水质。强化海洋生态保护，集约节约利用海域资源，加快发展海洋经济。深入实施蓝天工程，全面开展大气污染防治，推进五矿中板80兆瓦煤气发电以及营口钢铁煤气资源综合利用项目，不断提升我市环境空气质量。着力加强环境监管，坚决打好打胜污染治理攻坚战，建设山更绿、水更清、天更蓝的美好家园。

　　（五）着力保障改善民生，大力发展社会各项事业

　　坚持围绕发展抓民生，围绕民生抓发展，集中有限资金，补短板、抓急需，提高群众生活水平。

　　加大民生工程的实施力度。实施食品安全工程，提升食品和农产品质量安全监管能力；实施基础教育工程，促进基础教育均衡发展；实施养老服务体系提升工程，促进养老服务资源共享；实施城区道路畅通工程，改善市民出行环境；实施城市服务功能提升工程，促进城市生活便利化、城市文化形象化、城市环境生态化；实施美丽乡村工程，进一步改善农村生产、生活和生态环境；实施社会公益保障工程，拓展保障服务领域和受益面；实施东部振兴工程，持续改善山区群众生产生活条件。通过8项重点民生工程、35个具体项目的实施，增进人民福祉，让群众生活质量有一个较大幅度的提升。

　　加强就业和社会保障体系建设。全市新增实名制就业4.5万人，对困难家庭高校毕业生实施兜底安置，就业率达到100%；做好养老、医疗等保险扩面和基金征缴，全市城镇职工基本养老保险参保人数达到81.6万人，基本医疗保险参保人数达到118.6万人；适时提高城乡低保标准；加强社会救助，建立“一门受理、协同办理”的社会救助机制，实现城乡医疗救助“一站式”管理。

　　大力发展社会各项事业。加强农村义务教育薄弱学校改造，促进城乡教育均衡发展。适应高考改革，及时调整教学管理模式；稳妥做好营口一高中搬迁工作，高质量建设中等职业教育园区，新建营口高技能人才公共实训中心，完善营口理工学院办学的体制机制。加强公共卫生服务体系和农村基层医疗服务体系建设，提高新农合保障能力和水平。全面繁荣文化事业，推动群众文化活动扎根基层。完成辽河大剧院改造工程，加快市图书大厦和少儿图书馆新馆建设；大力发展乐器、文化创意等特色文化产业，壮大文化产业规模。开放学校体育场所，促进全民健身活动蓬勃开展。积极开展双拥共建活动，争取全国双拥模范城创建4连冠。

　　加强社会治理。开展“警务规范年”活动，不断提高执法公信力和打防管控水平。强化社会治安综合治理，完善智慧型社会治安防控体系，不断推进平安营口建设。努力创新社区治理体制机制，提升管理服务水平。依法推进信访制度改革，规范信访程序和秩序，提高信访工作质量。贯彻落实新《安全生产法》，强化责任落实，保持安全生产持续稳定。开展食品药品安全专项整治，确保人民群众饮食用药安全。

　　抓紧做好“十三五”规划编制工作。加强国防教育、民兵预备役建设；做好统计、物价监管、人民防空、人口和计划生育、新闻出版、国家安全、民族宗教、外事侨务、史志、档案、地震等工作；积极发展慈善、老龄、妇女儿童、残疾人、未成年人等各项事业。

　　（六）坚持依法行政，建设法治政府

　　深入学习贯彻十八届四中全会精神，加强法治教育，树立法治思维，强化法律意识，构建法治政府治理体系。

　　严格依法行政。坚持“法无授权不可为、法定职责必须为”，坚决纠正不作为、乱作为，坚决克服懒政、怠政，坚决惩处失职、渎职。严格规范公正文明执法，加大依法行政考评和监督检查力度，建立规范、顺畅、高效的行政秩序。深入推进政务公开，不断增强政府透明度和公信力。自觉接受人大依法监督和政协民主监督，认真办理人大代表建议和政协提案，努力建设职能科学、权责法定、执法严明、公开公正、廉洁高效、守法诚信的法治政府。

　　加强作风建设。扎实开展“解放思想，积极主动适应新常态，转变观念，坚定不移谋求新发展”大讨论活动，持之以恒抓好“四风”整治，巩固和拓展党的群众路线教育实践活动成果。增强责任意识，保持发展激情，大力倡导敢于担当、勇于作为的进取精神；大力弘扬只争朝夕、善做善成的务实作风，营造良好的政务环境。政府公务员要从自身做起、从小事做起，带头坚守正道、弘扬正气；坚持深入基层、走进群众，一心为公、忠诚为民，尽心竭力为人民服务、对人民负责、受人民监督、让人民满意。

　　加强诚信政府建设。政府做出的承诺必须兑现，上级做出的各项决策部署必须不折不扣地落到实处，做到政令畅通、令行禁止，以政府诚信引领个人诚信、企业诚信，全面建设诚信营口。

　　坚持廉洁从政。自觉履行“三严三实”准则，坚决做到讲诚信、懂规矩、守纪律，为政清廉、谨慎用权，敬法畏纪、遵规守矩。加强政府系统惩防腐败体系建设，强化行政监察和审计监督，严格执行党风廉政建设责任制。市政府班子要率先垂范，敢抓敢管、严抓严管，积极营造良好的政治生态和从政环境。

　　各位代表：站在营口老工业基地全面振兴的新起点，顺应人民群众过上更好生活的新期待，面对前所未有的新机遇，我们必须肩负起振兴发展的历史使命，切实担当起为民谋利的重大责任，在省委、省政府和市委的正确领导下，紧紧依靠全市人民，“崇尚实干，狠抓落实”，在营口科学发展、可持续发展、包容发展的道路上谱写新的辉煌篇章！
